# Serological testing of Schmallenberg virus in Swedish wild cervids from 2012 to 2016

**DOI:** 10.1186/s12917-017-1005-8

**Published:** 2017-04-04

**Authors:** A. Malmsten, J. Malmsten, G. Blomqvist, K. Näslund, C. Vernersson, S. Hägglund, A.-M. Dalin, E. O. Ågren, J.-F. Valarcher

**Affiliations:** 1 Department of Clinical Sciences, Division of Reproduction, Swedish University of Agricultural Sciences, Box 7054, 750 07 Uppsala, Sweden; 2 Department of Pathology and Wildlife Diseases, National Veterinary Institute, 751 89 Uppsala, Sweden; 3 Department of Wildlife, Fish, and Environmental studies, Swedish University of Agricultural Sciences, 901 83 Umeå, Sweden; 4 Department of Microbiology, National Veterinary Institute, 751 89 Uppsala, Sweden; 5 Department of Clinical Sciences, Host Pathogen Interaction Group, DOS, Swedish University of Agricultural Sciences, Box 7054, 750 07 Uppsala, Sweden; 6 Department of Clinical Sciences, Host Pathogen Interaction Group, Ruminant medicine, Swedish University of Agricultural Sciences, Box 7054, 750 07 Uppsala, Sweden

**Keywords:** SBV, Moose, Roe deer, Red deer, Fallow deer, Serology, Orthobunyavirus, Scandinavia, Wildlife

## Abstract

**Background:**

Schmallenberg virus (SBV) first emerged in Europe in 2011, and in Sweden in late 2012. The virus was still circulating in parts of Europe in 2015. In recent testing, the virus has not been detected in Swedish domestic animals, indicating that it is no longer circulating in Sweden. It is not known if the virus has circulated and is still circulating in Swedish wild cervid populations and whether wildlife can act as virus reservoirs. The aim of this study was to investigate whether SBV has circulated, and is still circulating among wild cervids in Sweden.

**Results:**

Ninety-two sera from moose (*Alces alces*, *n* = 22), red deer (*Cervus elaphus*, *n* = 15), fallow deer (*Dama dama*, *n* = 44), and roe deer (*Capreolus capreolus*, *n* = 11) were collected and analyzed for antibodies against SBV. The sampling occurred in the southern and middle part of Sweden during three time periods: 1) before the vector season in 2012, 2) after the vector season in 2012, and 3) after the vector season in 2015. Animals from periods 1 and 2 were of varying ages, whereas animals collected in period 3 were born after the vector season 2013. Animals from period 1 (*n* = 15) and 3 (*n* = 47) were seronegative, but, 53% (16 of 30) of animals from period 2 were seropositive, determined by SBV competitive ELISA. Samples from period 2 were additionally analyzed for SBV-neutralizing antibodies. Such antibodies were detected in 16/16 SBV-N-antibody-positive, 3/12 negative and 2/2 doubtful sera. The two tests were in accordance at SBV-neutralizing antibody titers of 1:32 or higher.

**Conclusion:**

Our results show that SBV circulated among wild cervids during the vector season of 2012. Three years later, no SBV-antibodies were detected in animals born after the vector season 2013. The likely absence of SBV circulation in Sweden, in contrast to other parts of Europe, might be explained by the annual occurrence of a vector-free season due to climate conditions. Interpretations are limited by the small sample-size, but the results suggest that the SBV competitive ELISA has high specificity but might have slightly lower sensitivity compared to a seroneutralization assay, when using samples from wild cervids.

## Background

When an emerging disease affects an animal or human population, there are often social, economic and welfare consequences. This was the case in 2011 and 2012, when Schmallenberg virus (SBV) was detected in Europe [1]. Initially, clinical signs were observed mainly in cattle, and included diarrhea, pyrexia, and a drop in milk yield. Infection during the first part of gestation was followed by stillbirths and birth defects in the offspring. No relation to any current and known circulating pathogen was identified [2]. Shortly thereafter, the causative agent was shown to be a novel *Orthobunyavirus* of the family *Bunyaviridae* [3] that is transmitted by biting midges (*Culicoides* spp.) [4]. The virus has the ability to cross the placental barrier and cause lesions in fetuses and neonates [5]. It infects a number of domestic and wild animal species including cattle, sheep, goats [6], alpaca [7] red deer, roe deer [8], fallow deer, moose, bison [9], wild boar [10], dogs [11], and a number of zoo animals [12]. In 2014 and 2015 the virus was still circulating in continental Europe [13, 14]. In Sweden, SBV was first detected in 2012 in domestic animals in the south. The virus spread rapidly north beyond the Arctic Circle, and occurred in high prevalence in tested animals [15]. However, it was not known if the virus did circulate (in 2012) or still is circulating in wildlife ruminant populations, and if they could act as reservoirs for the virus [16].

The aim of this study was to investigate if SBV is circulating among wild cervids in Sweden. Two hypotheses were tested: a) SBV-specific serum antibodies can be detected in Swedish wild cervids to the same extent and during the same time periods as SBV was diagnosed in domestic ruminants. b) SBV is still widely circulating in wild ruminant populations, despite likely being absent in the domestic ruminant population.

## Methods

### Sampling collection

Sera from moose (*Alces alces*, *n* = 22), red deer (*Cervus elaphus*, *n* = 15), fallow deer (*Dama dama*, *n* = 44), and roe deer (*Capreolus capreolus*, *n* = 11) were collected during three time periods: 1) before the vector season in 2012 (February, samples collected for biobanking), 2) after the vector season in 2012 (October 2012 - February 2013) and, 3) after the vector season in 2015 (November 2015 - January 2016, see Table 1 for species and sample distribution).

**Table 1:** Results of serological testing by cELISA of Swedish wild cervids for the detection of specific antibodies directed against Schmallenberg virus in different time periods

Species	Time period 1	Time period 2	Time period 3
	(Feb 2012)	(Nov 2012 - Feb 2013)	(Nov 2015 - Jan 2016)
	No. positive/ no. tested (%)	No. positive/ no. tested (%)	No. positive/ no. tested (%)
Moose	0/15 (0)	3/4 (75.0)	0/3 (0)
Roe deer		2/6 (33.3)	0/5 (0)
Red deer		0/4 (25.0)	0/11 (0)
Fallow deer		11/16 (75.0)	0/28 (0)
Total	0/15 (0)	16/30 (60.0)	0/47 (0)

In time period 1, captured live adult moose aged >2 years were sampled. In time periods 2 and 3, hunter-killed moose, red deer, fallow deer, and roe deer were sampled. The animals from time period 2 were of varying age. In time period 3 samples were collected from animals that were born after the vector season 2013 and aged between 0.5 to 1.5 years to avoid testing animals that could have been exposed to SBV in earlier time periods.

**Fig. 1:**
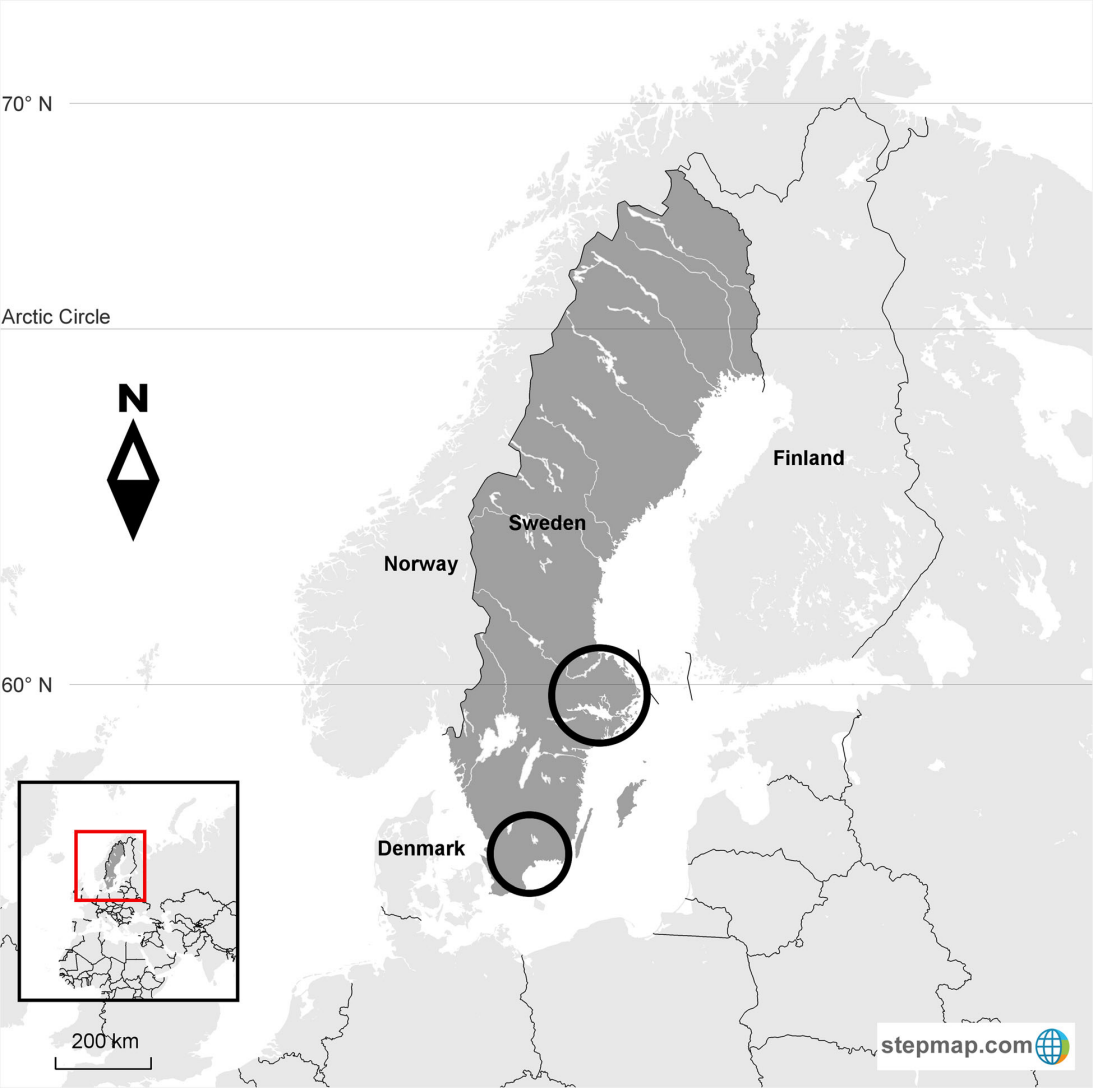
Map of Sweden with circles indicating sampling regions where Swedish wild cervids were sampled and tested for antibodies against Schmallenberg virus

Age in all sampled species was determined by investigating tooth eruption patterns, and antler development status. All sampling occurred in the southern and middle parts of Sweden (Fig. 1) where domestic ruminants previously had been tested positive for antibodies against SBV [15], whereas recent testing had shown negative results. Blood samples were collected in sterile dry tubes (BD Vacutainer®, Franklin Lakes, USA) kept at room temperature 24 h before centrifugation at 3000×g for ten minutes. The sera were stored in −20 °C prior to analysis.

### Serology

All sera were analyzed by competitive ELISA (cELISA, ID Screen® Schmallenberg virus Competition Multi-species) according to the manufacturer’s instructions [17]. This ELISA detects antibodies by competition with conjugated antibodies specific to the SBV nucleoprotein (N). The sera were tested undiluted in duplicate and results were expressed as competition percentage (S/N%), based on the mean optical density (OD)_Sample_/OD _Negative Control_ X100. As indicated in the instructions, sera with S/N% greater than 50% were considered as negative, >40-50% were classified as doubtful, and ≤40% as positive. A positive control provided with the kit was included in each run.

Sera collected during the initial SBV outbreak in Sweden [15] (time period 2) were additionally analyzed for SBV-neutralizing antibodies. The sera were heat-inactivated, 2-fold diluted from 1:8 to 1:512 and analyzed in duplicate. Fifty microliters of each dilution was mixed with an equal volume of EMEM containing 150 tissue culture infectious dose 50% (TCID_50_) SBV (BH80/11-4, kindly provided by the Friedrich-Loeffler Institute, Germany) and was incubated for one hour at 37 °C. Thereafter, approximately 20,000 BHK-21 cells in a volume of 50 μl in EMEM supplemented with 20% fetal calf serum were added to each well. The plates were then incubated for 3-4 days at 37 °C under 5% CO_2_. The cells were examined daily in a light microscope for the presence of SBV-specific cytopathogenic effects (cpe). The neutralizing titer of a serum was determined as the highest dilution in which the cell monolayer was intact. A serum sample was considered negative if cpe was observed at and from a dilution of 1:8 [18, 19].

## Results

All analyzed samples collected from wild cervids before the vector season 2012 (*n* = 15) as well as those collected after the vector season 2015 animals (*n* = 47, born after the vector season 2013) were seronegative for SBV by cELISA (Table 1). In sera collected during time period 2 after the vector season 2012, SBV-N-specific antibodies were detected by cELISA in 16 sera out of 30 (53%) (Table 1). One red deer and one fallow deer showed doubtful reaction in cELISA (Table 2).

**Table 2:** Sera from wild cervids sampled in Sweden between November 2012 and February 2013, and tested for SBV antibodies by serum neutralization test (SNT), and competitive ELISA (cELISA)

SNT		cELISA		
Titer	No	No positive (≤40%)	No doubtful (>40-50%)	No negative (>50%)
<8	9 (3 red deer, 3 fallow deer, 3 roe deer)	-	-	9 (3 red deer, 3 fallow deer, 3 roe deer)^a^
8	7 (1 red deer, 4 fallow deer, 2 roe deer)	4 (3 fallow deer, 1 roe deer)^a^	1 (1 red deer)	2 (1 fallow deer, 1 roe deer)
16	6 (1 moose, 4 fallow deer, 1 roe deer)	4 (3 fallow deer, 1 roe deer)^a^	1 (1 fallow deer)	1 (1 moose)
32	3 (1 moose, 2 fallow deer)	3 (1 moose, 2 fallow deer)^a^	-	-
64	5 (2 moose, 3 fallow deer)	5 (2 moose, 3 fallow deer)^a^	-	-

^a^Indicates agreement of the results of the two tests

Sera from time period 2 were additionally analyzed for SBV-neutralizing antibodies. Such antibodies were detected in 16/16 SBV-N-antibody-positive sera, in 3/12 SBV-N-antibody negative and 2/2 doubtful sera. No SBV-specific antibodies were detected by any of the techniques in nine animals (Table 2). Seropositive animals were found in all sampling areas and in all tested cervid species (moose, roe deer, red deer, and fallow deer). The SBV-neutralizing antibody titers varied between species (Table 2), but low sample sizes precluded statistical analyzes with regards to inter- and intraspecies titer variation. These data show a very good agreement between both tests for negative samples or when the SBV-neutralizing antibody titers were >1/16. Five samples with an SBV-neutralizing antibody titer of 1/8 (*n* = 3) or 1/16 (*n* = 2) were found doubtful (*n* = 2) or negative (*n* = 3) by cELISA.

## Discussion

This is the first report of findings of SBV-specific antibodies in Scandinavian wildlife, showing that wild cervids in Sweden were exposed to the virus in the summer of 2012 and probably not after the vector season 2014. Moreover, this study indicates that SBV infection in wildlife and domesticated animals follows the same pattern in the same area in which cattle were seropositive in 2012 and 2013 but seronegative in a recent serological survey when born after the vector season 2013 (S. Zohari, personal communication). Schmallenberg virus does not seem to circulate in Sweden anymore, in contrast to other European countries such as Germany [13]. The difference between Sweden and countries in central and southern Europe is that Sweden has a long vector-free season. Virus transmission and spread is possible at temperatures around 15 °C with a temperature optimum between 18 °C and 19 °C due to vector limitations [20]. In Sweden, such daily mean temperatures are usually limited to May-August [21], but may occur in September as well. Virus persistence depends on the winter survival of adult midges which must have access to a naïve ruminant population. The absence of SBV antibodies in the tested animals suggests that the virus does not persist in ruminants (wild or domestic) or in the environment during the vector-free season. However, to completely verify that the infection is not present in Swedish cervid populations, a larger and predetermined number of animals need to be tested.

A seroneutralization test (SNT) was used at first to test wildlife following the emergence of SBV in Europe, since no cELISA was available to measure specific antibodies against SBV. Since then, a cELISA has been developed with high sensitivity and specificity for domestic ruminant samples [17], and in addition, is easier to perform. Our results indicate that the two methods are in agreement for most samples tested and the level of agreement increases with increasing titer. It is possible that none of the techniques perform well for samples with low titers. Our data suggest that the cELISA lacks sensitivity for sera with low antibody titers. The SNT, on the other hand, might lack specificity for sera at low dilution, as previously observed for sheep [22]. Nevertheless, in contrast to in the sera collected between November 2012 and February 2013, no specific antibodies were detected by cELISA in sera collected in 2015 from animals aged between 0.5 and 1.5 years of age, which suggest that the SBV has not recently circulated among wild cervids in the investigated area.

The effect of SBV in wildlife is still unknown. No clinical signs of SBV were reported in Swedish wildlife during the summer and early autumn of 2012 when the outbreak was detected. Possible signs of SBV infection in Swedish wild or fenced cervids, in the form of abortions and congenital malformations, were not reported during 2013 [23]. However, it is unlikely to make such observations in wild animals for a number of reasons such as scavenging birds and mammals. Furthermore, it requires a primary maternal infection and viremia during a particular period of pregnancy, i.e. after the first placentoma has developed and before the fetuses are immunocompetent [24]. The circulation of SBV most likely did not coincide with the placentoma formation in wild cervids in Sweden. Hence, delayed consequences of SBV infection in Swedish cervids are presumed to be minimal.

## Conclusion

Schmallenberg virus was circulating among Swedish wild cervids during the vector season 2012, but no serological evidence of SBV was found during subsequent testing in 2015 and 2016. Based on Swedish climate conditions and the results of this study, we can assume that Sweden has an unfavorable climate for SBV-overwintering vectors. This might have contributed to the fact that the virus infection has seemingly not become endemic or possibly not reoccurred in Sweden. Midge activity and the reproductive season of Swedish wild cervids, is a seasonal and biological mismatch for the virus, which may explain that SBV has little impact on Swedish wild ruminant health. These animals are thus highly unlikely to be reservoirs of this virus.

## Abbreviations

cELISA: Competitive enzyme-linked immunosorbent assay
CPE: Cytopathogenic effects
N: Nucleoprotein
OD: Optical density
SBV: Schmallenberg virus
SNT: Seroneutralization test
TCID: Tissue culture infectious dose
